# Association between community-level social trust and the risk of dementia: A retrospective cohort study in the Republic of Korea

**DOI:** 10.3389/fpubh.2022.913319

**Published:** 2022-10-06

**Authors:** Jaeyi Hong, Sun Jae Park, Jong-Koo Lee, Hye Jin Jeong, Juhwan Oh, Seulggie Choi, Seogsong Jeong, Kyae Hyung Kim, Joung Sik Son, Sang Min Park

**Affiliations:** ^1^Department of Biomedical Sciences, Seoul National University Hospital, Seoul National University College of Medicine, Seoul, South Korea; ^2^Department of Statistics, University of Illinois at Urbana-Champaign, Champaign, IL, United States; ^3^National Academy of Medicine of Korea, Seoul, South Korea; ^4^Department of Family Medicine, Seoul National University Hospital, Seoul National University College of Medicine, Seoul, South Korea; ^5^Comprehensive Care Clinic, Public Healthcare Center, Seoul National University Hospital, Seoul, South Korea; ^6^Department of Medicine, Seoul National University College of Medicine, Seoul, South Korea; ^7^Department of Internal Medicine, Seoul National University Hospital, Seoul National University College of Medicine, Seoul, South Korea; ^8^Department of Biomedical Informatics, CHA University School of Medicine, Seongnam, South Korea; ^9^Department of Internal Medicine, Hallym University Sacred Heart Hospital, Anyang, South Korea

**Keywords:** community-level social trust, dementia, cohort study, Alzheimer's disease, Republic of Korea

## Abstract

**Introduction:**

It is known that biological risk factors and lifestyle behaviors are important determinants of dementia. However, there has been yet to be sufficient evidence to prove that community-level social capital is one of the determinants of dementia. This retrospective cohort study is a large, long-term, population-based study that investigated the association between community-level social trust and the risk of dementia in the Republic of Korea.

**Methods:**

Data came from the Korean National Health Insurance Service database. The community-level social trust values were determined by the Korean Community Health Survey. The study population consisted of 1,974,944 participants over 50 years of age and was followed up from 1 January 2012 to 31 December 2019 with a latent period of 5 years from 1 January 2012 to 31 December 2016. Cox proportional hazards regression was utilized to obtain the adjusted hazard ratios (aHRs) and 95% confidence intervals (CIs) for the risk of dementia according to social trust quintiles.

**Results:**

Participants within the highest quintile of community-level social trust had a lower risk for overall dementia (aHR, 0.90; 95% CI, 0.86–0.94) and Alzheimer's disease (aHR, 0.90; 95% CI, 0.85–0.94) compared to those within the lowest quintile of community-level social trust. The alleviating trend association of high community-level social trust on dementia risk was maintained regardless of whether the participants had health examinations.

**Conclusions:**

Our findings suggest that higher community-level social trust is associated with a reduced risk of dementia. Community-level social trust is a crucial indicator of dementia and improving community-level social trust may lead to a lower risk of dementia.

## Introduction

Dementia is a disease with high prevalence and high societal burden (1). In 2019, there were 57.4 (95% CI 50.4–65.1) million cases of dementia worldwide (2). By 2050, the Republic of Korea is expected to have 3.02 million cases of dementia among people over 65 years of age, up from 830,000 cases in 2020 (3). The typical risk factors of dementia are biological factors and lifestyle behaviors. Previous studies have demonstrated that community-level social capital is one of the determinants of dementia, but there has yet to be sufficient evidence to prove that such is the case (4, 5).

Social capital can be broadly defined as a “collective asset in the form of shared norms, values, beliefs, trust, networks, social relations, and institutions that facilitate cooperation and collective action for mutual benefits” (6). While the definition of social capital can be controversial, it can be distinguished into two main types: structural and cognitive dimensions. Structural dimensions are the external behaviors of individuals within the groups and may include “networks, relationships, and institutions” (7). On the other hand, cognitive dimensions are the perceptions of individuals “of the level of interpersonal trust and norms of reciprocity within the group” (8) and may include “values, attitudes, trust, confidence, and norms” (7). Both dimensions may be conceived or studied on either individual or collective levels (8).

Among the various cognitive dimensions of social capital, social trust has been recognized as an important factor on people's health outcomes. Subramanian et al. (9) investigated the association between social trust and self-rated health in the United States. The study found that higher community social trust levels led to a lower probability of poor health reports (9). In addition, Kuroki (10) in Japan showed that social trust is causally associated with individual happiness. Additionally, one study studied a relationship between community-level social capital, including community-level social trust, and sexual behaviors on adolescents in Tanzania (11). As investigated in previous studies, individual and community-level social trust are both influential on health outcomes. However, our study focused on community-level social trust because trust is a collective feature of groups, typically conceptualized by aggregating individual responses of people (12), and may be able to independently impact health outcomes.

There are other studies from the Republic of Korea which also investigated the association between community-level social trust and various health outcomes such as metabolic syndrome (MetS), cardiovascular disease (CVD), and mortality. In those studies, higher community-level social trust was associated with better health status (13–15). Meanwhile, there is limited research regarding the association of community-level social trust with the risk of dementia in the Republic of Korea. One study from the American Association for Geriatric Psychiatry found that cognitive decline may be a pathological marker of Alzheimer's disease, the most common type of dementia (16), highlighting the importance of studying a relationship between cognitive factors and dementia. In this respect, it is crucial to investigate the relationship between community-level social trust, a cognitive dimension of social capital, and the risk of dementia–thus calling for a need to investigate the association between community-level social trust and the risk of dementia among a large population in the Republic of Korea.

## Methods

### Data source

The study population was obtained from the Korean National Health Insurance Service (NHIS) database (NHIS-2021-1-755). NHIS is an institution that provides compulsory health insurance and corresponding services for all Korean citizens (17). For all relevant individuals aged 40 years or older, NHIS further offers health screening examinations that consist of a self-reported questionnaire on health-related behaviors, anthropometric measurements, and laboratory tests for blood and urine. For claims purposes, the NHIS congregates health service exploitation information on all insured health services, including inpatient and outpatient hospitalizations, health screening examinations, diagnostic and treatment-related procedures, and pharmaceutical prescriptions. Several previous extensive epidemiological studies utilizing the NHIS database have been studied, and its validity can be accounted for (17).

### Study population

**Figure 1:**
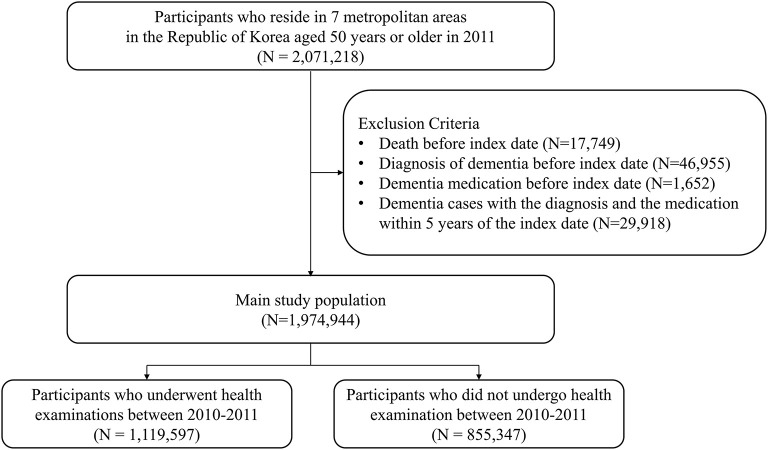
Study sample selection.

Study sample selection is presented in Figure 1. Among 2,071,218 initial participants from seven metropolitan areas in the Republic of Korea aged 50 years or older in 2011, 17,749 participants were excluded as they passed away before 2012, and 46,955 participants with the diagnosis of dementia before the index date of 1 January 2012 were excluded. 1,652 participants were also excluded as they were prescribed medication for dementia before the index date. Additionally, 29,918 participants were excluded because of dementia cases with the diagnosis and medication within 5 years of the index date. This led the main study population to consist of 1,974,944 participants who followed up on their diagnosis and prescription for dementia to define incident dementia from 1 January 2012 to 31 December 2019. Among the main study population, 1,119,597 participants underwent health examinations between 2010 and 2011, and 855,347 participants did not.

### Key variables

We obtained community-level social trust values based on the Korean Community Health Survey (KCHS) (18), coordinated by the Korea Disease Control and Prevention Agency in 2011. KCHS is a nationally representative community-based cross-sectional survey with community-level information corresponding to administrative district areas. All survey participants were asked, “Do you trust members of your community?” as a social trust measure (19). The proportion of participants who answered “yes” to each administrative district area's social trust question was utilized to calculate the social trust value. The social trust values in 2011 were combined according to each participant's area of residence, which is also assigned to the same administrative district area. Study participants were divided into quintiles based on social trust values, with the lowest social trust values appearing in the first quintile.

The attending physician has a duty to put the International Classification of Diseases, Tenth Revision (ICD-10) codes for the primary cause of hospitalization upon hospital admission. Dementia was measured using both medications (tacrine, donepezil, rivastigmine, and galantamine) and diagnosis for dementia (ICD-10 code F00, F01, F02, F03, or G30), Alzheimer's disease (AD; ICD-10 code F00, G30) and vascular dementia (VD; ICD-10 code F01) (20). Since dementia is a slowly progressing disease over time, all analyses were conducted based on a 5-year wash-out period that did not consider new cases of dementia diagnosis from 1 January 2012 to 31 December 2016.

Potential confounders including age in years (continuous), sex (categorical, men or women), categorical area of residence (capital-Seoul, or metropolitan city–Busan, Daegu, Incheon, Gwangju, Daejeon, and Ulsan), categorical household income in first, second, third, and fourth quartiles, and Charlson comorbidity index (continuous) were adjusted. The household income was calculated from the insurance premium. The individual-level confounding variables such as depression diagnosis (categorical, no or yes), diabetes diagnosis (categorical, no or yes), or cardiovascular disease diagnosis (categorical, no or yes) before 1 January 2012 were adjusted additionally.

### Statistical analysis

As criteria for determining the qualities of the study population, the Chi-squared test was exploited for categorical variables, and the analysis of variance was used for continuous variables. For dementia risk in accordance with social trust quintiles, the adjusted hazard ratios (aHRs) and 95% confidence intervals (CIs) were calculated using multivariate Cox proportional hazards regression. The relationship between social trust and the risk of dementia was stratified according to age, sex, household income, Charlson comorbidity index, depression, cardiovascular disease, and diabetes. We also conducted analyses of the risk of dementia according to social trust considering participation in health examinations. Finally, we provided the aHRs per one interquartile range increase of the community-level social trust to show that the results align with the result from the adjusted hazard ratios using quintiles of the community-level social trust values.

We defined statistical significance as a *p*-value of <0.05 on a two-sided basis. The data collection and statistical analysis were carried out using SAS 9.4 (SAS Institute, Cary, NC, USA).

## Results

Table 1 summarizes the descriptive qualities of the study population. The ranges for social trust were 41.54–52.83, 53.08–58.32, 58.33–60.20, 60.21–67.62, and 67.92–88.46%. Social trust in the highest quintile was associated with more metropolitan residents, a higher income, and fewer comorbidities (all *p*-values < 0.001). The number of participants who previously had depression, cardiovascular disease, or diabetes was also measured, and there were significant differences in the distribution of all confounders (all *p*-values < 0.001).

**Table 1:** Descriptive characteristics of the main study population.

	**Social trust (Quintiles)**					***P*-value**
	**1st (Lowest)**	**2nd**	**3rd**	**4th**	**5th (Highest)**	
Range, %	41.54–52.83	53.08–58.32	58.33–60.20	60.21–67.62	67.92–88.46	
Number of people	394,114	383,687	407,270	398,640	391,233	
Age, years, mean (SD)	55.53 (10.49)	55.23 (10.25)	55.52 (10.53)	56.00 (10.62)	55.24 (10.44)	<0.001
**Sex**, ***N*** **(%)**						<0.001
Men	190,207 (48.26)	184,018 (47.96)	195,833 (48.08)	191,379 (48.01)	189,254 (48.37)	
Women	203,907 (51.74)	199,669 (52.04)	211,437 (51.92)	207,261 (51.99)	201,979 (51.63)	
**Area of residence**, ***N*** **(%)**						<0.001
Capital	243,157 (61.70)	156,964 (40.91)	267,368 (65.65)	142,960 (35.86)	55,666 (14.23)	
Metropolitan city	150,957 (38.30)	226,723 (59.09)	139,902 (34.35)	255,680 (64.14)	335,567 (85.77)	
**Household income, quartiles**, ***N*** **(%)**						<0.001
1st (highest)	127,115 (32.25)	136,461 (35.57)	154,668 (37.98)	137,046 (34.38)	151,115 (38.63)	
2nd	91,330 (23.17)	86,839 (22.63)	86,818 (21.32)	90,877 (22.80)	87,420 (22.34)	
3rd	79,290 (20.12)	70,348 (18.33)	70,159 (17.23)	72,097 (18.09)	64,666 (16.53)	
4th (lowest)	96,379 (24.45)	90,039 (23.47)	95,625 (23.48)	98,620 (24.74)	88,032 (22.50)	
**Charlson comorbidity index**, ***N*** **(%)**						<0.001
0	76,235 (19.34)	72,063 (18.78)	78,797 (19.35)	73,157 (18.35)	70,357 (17.98)	
1	86,823 (22.03)	86,304 (22.49)	90,559 (22.24)	85,542 (21.46)	87,265 (22.31)	
≥2	231,056 (58.63)	225,320 (58.72)	237,914 (58.42)	239,941 (60.19)	233,611 (59.71)	
**Previous depression**, ***N*** **(%)**						<0.001
No	358,761 (91.03)	348,171 (90.74)	368,796 (90.55)	360,923 (90.54)	354,155 (90.52)	
Yes	35,353 (8.97)	35,516 (9.26)	38,474 (9.45)	37,717 (9.46)	37,078 (9.48)	
**Previous cardiovascular disease**, ***N*** **(%)**						<0.001
No	318,912 (80.92)	311,196 (81.11)	327,778 (80.48)	318,616 (79.93)	319,818 (81.75)	
Yes	75,202 (19.08)	72,491 (18.89)	79,492 (19.52)	80,024 (20.07)	71,415 (18.25)	
**Previous diabetes**, ***N*** **(%)**						<0.001
No	307,341 (77.98)	303,645 (79.14)	320,770 (78.76)	308,730 (77.45)	309,899 (79.21)	
Yes	86,773 (22.02)	80,042 (20.86)	86,500 (21.24)	89,910 (22.55)	81,334 (20.79)	

P-value calculated by Chi-squared test for categorical variables and analysis of variance for continuous variables. SD, standard deviation; N, number of people.

Table 2 depicts the association of community-level social trust with dementia. Compared to those in the lowest quintile of social trust, those in the highest quintile had a lower risk for overall dementia (aHR, 0.90; 95% CI, 0.86–0.94). There was an alleviating trend with higher levels of social trust for overall dementia. Participants in the highest social trust quintile experienced a lower risk of Alzheimer's disease than those in the lowest social trust quintile (aHR, 0.90; 95% CI, 0.85–0.94). Though the results were not statistically significant, vascular dementia showed a similar trend to overall dementia or Alzheimer's disease. The trend was unchanged when the third quintile of social trust value group was the reference group. The risk of dementia was the lowest in the highest social trust quintile group for overall dementia (aHR, 0.92; 95% CI, 0.88–0.96), Alzheimer's disease (aHR, 0.93; 95% CI, 0.89–0.98), and vascular dementia (aHR, 0.87; 95% CI, 0.75–1.004). Supplementary Table S1 shows the adjusted hazard ratio for dementia per 1 Interquartile Range (IQR) increase in the social trust index. As social trust index increased by 1 IQR value, the risk of overall dementia (aHR, 0.98; 95% CI, 0.96–0.99; *p*-value, 0.005) and Alzheimer's disease (aHR, 0.98; 95% CI, 0.96–0.99; *p-*value, 0.003) decreased.

**Table 2:** Hazard ratios for dementia according to social trust.

	**Social trust (Quintiles)**				
	**1st (Lowest)**	**2nd**	**3rd**	**4th**	**5th (Highest)**
Number of people	394,114	383,687	407,270	398,640	391,233
**Overall dementia**
Events	5,040	4,606	5,391	5,675	5,127
Person-years	3,048,525	2,975,285	3,155,512	3,077,398	3,030,278
aHR (95% CI)^a^	1.00 (reference)	0.91 (0.87–0.95)	0.98 (0.94–1.02)	0.95 (0.91–0.99)	0.90 (0.86–0.94)
aHR (95% CI)^b^	1.02 (0.98–1.07)	0.93 (0.89–0.97)	1.00 (reference)	0.97 (0.93–1.01)	0.92 (0.88–0.96)
**Alzheimer's disease**
Events	4,476	4,151	4,790	5,092	4,661
Person-years	3,049,351	2,975,958	3,156,393	3,078,293	3,030,984
aHR (95% CI)^a^	1.00 (reference)	0.91 (0.87–0.95)	0.96 (0.92–1.01)	0.94 (0.90–0.98)	0.90 (0.85–0.94)
aHR (95% CI)^b^	1.04 (0.99–1.09)	0.95 (0.91–0.99)	1.00 (reference)	0.98 (0.94–1.02)	0.93 (0.89–0.98)
**Vascular dementia**
Events	511	381	516	518	453
Person-years	3,054,289	2,980,716	3,161,951	3,084,016	3,036,179
aHR (95% CI)^a^	1.00 (reference)	0.80 (0.70–0.93)	1.03 (0.90–1.18)	0.98 (0.85–1.13)	0.90 (0.77–1.05)
aHR (95% CI)^b^	0.97 (0.85–1.11)	0.78 (0.68–0.90)	1.00 (reference)	0.95 (0.83–1.08)	0.87 (0.75–1.004)

The aHRs were calculated by Cox proportional hazards regression after adjustments for age, sex, area of residence, household income, Charlson comorbidity index, depression, cardiovascular disease, and diabetes. aHR (95% CI)^*a*^: First quintile group is the reference group for all adjusted hazard ratios. aHR (95% CI)^*b*^: Third quintile group is the reference group for all adjusted hazard ratios. aHR, adjusted hazard ratio; CI, confidence interval.

Table 3 illustrates the association between social trust and dementia according to age, sex, household income, and Charlson comorbidity index. In most of the stratification, consistent associations were discovered. Among participants with age <65 years (aHR, 0.89; 95% CI, 0.77–0.995) or ≥65 years (aHR, 0.92; 95% CI, 0.87–0.97), participants in the highest quintile of social trust had a lower risk of overall dementia. Similarly, lower overall dementia was seen among men (aHR, 0.88; 95% CI, 0.81–0.95) as well as women (aHR, 0.91; 95% CI, 0.86–0.96) within the highest social trust quintile. In most subgroups divided by household income and Charlson comorbidity index, an alleviating association for those with higher social trust on overall dementia was discovered. The stratified results on the relationship between social trust and dementia according to depression, cardiovascular disease, and diabetes are represented in Supplementary Table S2. After the stratified analysis, the risk-reducing trend of high social trust in dementia was maintained.

**Table 3:** Stratified analysis on the association of social trust with dementia according to age, sex, household income, and Charlson comorbidity index.

	**aHR (95% CI)**					
	**Social trust (Quintiles)**					***P*-value for trend**
	**1st (Lowest)**	**2nd**	**3rd**	**4th**	**5th (Highest)**	
**Overall dementia**
**Age, years**
<65	1.00 (reference)	0.82 (0.73–0.91)	0.99 (0.89–1.10)	0.90 (0.79–1.01)	0.89 (0.77–0.995)	0.841
≥65	1.00 (reference)	0.95 (0.91–1.001)	0.995 (0.95–1.04)	0.97 (0.92–1.01)	0.92 (0.87–0.97)	0.019
**Sex**
Men	1.00 (reference)	0.93 (0.86–0.998)	0.95 (0.89–1.02)	0.98 (0.91–1.05)	0.88 (0.81–0.95)	0.044
Women	1.00 (reference)	0.90 (0.85–0.95)	0.99 (0.94–1.05)	0.93 (0.88–0.99)	0.91 (0.86–0.96)	0.056
**Household income**
Upper half	1.00 (reference)	0.92 (0.87–0.98)	1.03 (0.97–1.09)	0.98 (0.92–1.03)	0.92 (0.87–0.98)	0.314
Lower half	1.00 (reference)	0.90 (0.84–0.96)	0.92 (0.86–0.98)	0.92 (0.86–0.98)	0.86 (0.80–0.93)	0.002
**Charlson comorbidity index**
0	1.00 (reference)	0.79 (0.67–0.93)	0.98 (0.84–1.15)	0.86 (0.73–1.02)	0.83 (0.70–0.99)	0.245
1	1.00 (reference)	0.85 (0.74–0.97)	1.03 (0.91–1.16)	0.94 (0.82–1.07)	0.90 (0.78–1.03)	0.528
≥ 2	1.00 (reference)	0.93 (0.88–0.98)	0.97 (0.93–1.02)	0.96 (0.91–1.01)	0.90 (0.86–0.95)	0.013
**Alzheimer's disease**
**Age, years**
<65	1.00 (reference)	0.80 (0.71–0.90)	0.94 (0.83–1.05)	0.89 (0.79–1.01)	0.86 (0.76–0.98)	0.498
≥65	1.00 (reference)	0.96 (0.91–1.01)	0.98 (0.94–1.03)	0.96 (0.91–1.004)	0.92 (0.88–0.98)	0.025
**Sex**
Men	1.00 (reference)	0.93 (0.86–1.002)	0.94 (0.87–1.01)	0.96 (0.89–1.04)	0.87 (0.80–0.95)	0.038
Women	1.00 (reference)	0.90 (0.85–0.96)	0.97 (0.92–1.03)	0.92 (0.87–0.98)	0.91 (0.86–0.97)	0.047
**Household income**
Upper half	1.00 (reference)	0.92 (0.86–0.98)	1.01 (0.95–1.07)	0.97 (0.91–1.03)	0.93 (0.87–0.99)	0.384
Lower half	1.00 (reference)	0.90 (0.84–0.97)	0.90 (0.84–0.97)	0.90 (0.84–0.97)	0.86 (0.79–0.93)	<0.001
**Charlson comorbidity index**
0	1.00 (reference)	0.81 (0.67–0.96)	1.02 (0.87–1.21)	0.84 (0.70–0.996)	0.82 (0.68–0.996)	0.209
1	1.00 (reference)	0.86 (0.75–0.99)	1.04 (0.91–1.19)	0.95 (0.83–1.09)	0.91 (0.78–1.05)	0.648
≥ 2	1.00 (reference)	0.93 (0.88–0.98)	0.94 (0.90–0.99)	0.94 (0.90–0.99)	0.90 (0.85–0.95)	0.008
**Vascular dementia**
**Age, years**
<65	1.00 (reference)	0.74 (0.56–0.997)	1.12 (0.85–1.47)	0.85 (0.63–1.14)	0.99 (0.73–1.35)	0.241
≥65	1.00 (reference)	0.84 (0.71–0.995)	1.02 (0.87–1.19)	1.03 (0.88–1.21)	0.88 (0.73–1.05)	0.973
**Sex**
Men	1.00 (reference)	0.92 (0.75–1.14)	1.01 (0.82–1.24)	1.05 (0.86–1.30)	0.95 (0.76–1.20)	0.778
Women	1.00 (reference)	0.72 (0.59–0.87)	1.05 (0.87–1.25)	0.92 (0.76–1.11)	0.85 (0.69–1.05)	0.757
**Household income**
Upper half	1.00 (reference)	0.80 (0.65–0.97)	1.09 (0.91–1.31)	0.94 (0.78–1.13)	0.88 (0.72–1.08)	0.839
Lower half	1.00 (reference)	0.81 (0.65–1.01)	0.95 (0.77–1.17)	1.04 (0.84–1.27)	0.92 (0.73–1.16)	0.656
**Charlson comorbidity index**
0	1.00 (reference)	0.54 (0.32–0.91)	0.50 (0.31–0.83)	0.78 (0.49–1.24)	0.72 (0.43–1.22)	0.533
1	1.00 (reference)	0.80 (0.53–1.20)	1.04 (0.71–1.53)	0.87 (0.58–1.32)	0.94 (0.61–1.45)	0.814
≥2	1.00 (reference)	0.84 (0.72–0.99)	1.10 (0.95–1.29)	1.03 (0.88–1.20)	0.92 (0.77–1.09)	0.549

The aHRs were calculated by Cox proportional hazards regression after adjustments for age, sex, area of residence, household income, Charlson comorbidity index, depression, cardiovascular disease, and diabetes. aHR, adjusted hazard ratio; CI, confidence interval.

Table 4 demonstrates the risk of dementia according to social trust among participants who received health examinations and who did not receive health examinations. Among the final study population, 1,119,597 participants underwent health examinations. After additional adjustments for dementia risk factors, participants in the highest quintile of social trust were at a lower risk of overall dementia (aHR, 0.93; 95% CI, 0.87–0.996) and Alzheimer's disease (aHR, 0.91; 95% CI, 0.85–0.98) than those in the lowest quintile of social trust. A similar trend was maintained when the reference group was changed to the third quintile of the social trust values. Among participants who did not undergo health examinations (855,347 participants in total), reduced risks of overall dementia (aHR 0.87; 95% CI, 0.81–0.93), Alzheimer's disease (aHR 0.88; 95% CI, 0.82–0.95), and vascular dementia (aHR, 0.76; 95% CI, 0.61–0.94) were observed when the first quintile of social trust was the reference group. The trend was unchanged when the third quintile of social trust was used as a reference group. A description of the qualities of participants who had health examinations is shown in Supplementary Table S3, and a description of the characteristics of individuals who did not undergo health examinations is represented in Supplementary Table S4.

**Table 4:** Risk for dementia according to social trust considering participation in health examinations.

			**Social trust (Quintiles)**				
	**Total**	**Events**	**1st (Lowest)**	**2nd**	**3rd**	**4th**	**5th (Highest)**
**Participants who underwent health examinations**							
**Overall dementia**
aHR (95% CI)^a^	1,119,597	13,044	1.00 (reference)	0.94 (0.88–0.996)	1.01 (0.95–1.07)	0.95 (0.89–1.01)	0.93 (0.87–0.996)
aHR (95% CI)^b^	1,119,597	13,044	0.99 (0.93–1.06)	0.93 (0.88–0.99)	1.00 (reference)	0.95 (0.90–0.999)	0.93 (0.87–0.98)
**Alzheimer's disease**
aHR (95% CI)^a^	1,119,597	11,673	1.00 (reference)	0.92 (0.86–0.99)	0.97 (0.90–1.03)	0.93 (0.87–0.999)	0.91 (0.85–0.98)
aHR (95% CI)^b^	1,119,597	11,673	1.04 (0.97–1.11)	0.95 (0.90–1.02)	1.00 (reference)	0.97 (0.91–1.03)	0.95 (0.89–1.01)
**Vascular dementia**
aHR (95% CI)^a^	1,119,597	1,191	1.00 (reference)	0.92 (0.74–1.13)	1.23 (1.01–1.49)	1.01 (0.82–1.24)	1.08 (0.86–1.34)
aHR (95% CI)^b^	1,119,597	1,191	0.82 (0.67–0.995)	0.75 (0.62–0.91)	1.00 (reference)	0.82 (0.69–0.99)	0.88 (0.72–1.07)
**Participants who did not undergo health examinations**							
**Overall dementia**
aHR (95% CI)^a^	855,347	12,795	1.00 (reference)	0.90 (0.84–0.95)	0.95 (0.90–1.01)	0.95 (0.89–1.01)	0.87 (0.81–0.93)
aHR (95% CI)^b^	855,347	12,795	1.05 (0.99–1.11)	0.94 (0.88–0.996)	1.00 (reference)	0.99 (0.94–1.05)	0.91 (0.85–0.97)
**Alzheimer's disease**
aHR (95% CI)^a^	855,347	11,497	1.00 (reference)	0.91 (0.85–0.97)	0.96 (0.90–1.02)	0.94 (0.88–0.999)	0.88 (0.82–0.95)
aHR (95% CI)^b^	855,347	11,497	1.05 (0.98–1.11)	0.95 (0.89–1.01)	1.00 (reference)	0.98 (0.92–1.04)	0.92 (0.86–0.98)
**Vascular dementia**
aHR (95% CI)^a^	855,347	1,188	1.00 (reference)	0.72 (0.59–0.88)	0.89 (0.74–1.07)	0.97 (0.80–1.17)	0.76 (0.61–0.94)
aHR (95% CI)^b^	855,347	1,188	1.13 (0.93–1.36)	0.81 (0.67–0.995)	1.00 (reference)	1.09 (0.91–1.30)	0.85 (0.69–1.06)

The aHRs were calculated by Cox proportional hazards regression after adjustments for age, sex, area of residence, household income, Charlson comorbidity index, depression, cardiovascular disease, and diabetes. aHR (95% CI)^*a*^: First quintile group is the reference group for all adjusted hazard ratios. aHR (95% CI)^*b*^: Third quintile group is the reference group for all adjusted hazard ratios. aHR, adjusted hazard ratio; CI, confidence interval.

## Discussion

In this large, long-term, population-based retrospective cohort study, higher community-level social trust was related to a lower risk of dementia. Among the three categories of dementia–overall dementia, Alzheimer's disease, and vascular dementia–the risk of overall dementia and Alzheimer's disease was reduced with higher community-level social trust. Even after additional adjustments with risk factors such as comorbidities, depression, diabetes, and cardiovascular disease, the results were preserved and statistically significant. Based on our information, this is the first countrywide cohort study to show an association of community-level social trust with dementia risk for an extensive general population worldwide.

Previously, other studies explored the association of various components of social capital, including social relationships, support, and community engagement with dementia. Kuiper et al. (21) performed a meta-analysis of longitudinal cohort studies on the association of social relationships with the risk of dementia. They concluded that social relationship factors that characterize a lack of social interaction are related to incident dementia (21). Since the study only included two studies from Asia, it is yet unclear about the relationship between social capital and incident dementia among the Asian population. However, our study provides significant results proving that there may be a negative association between high community-level social capital (trust aspect) and a lower risk of dementia using large population-based data. Also, a longitudinal study in Australia showed that low social support is associated with incident dementia (22). The study investigated both genders, but the results were only significant among women [Hazard Ratio (HR) = 1.79]. In contrast, the results in our study were not gender-specific, meaning the results were similar for men and women. Moreover, an English national cohort study presented that community engagement may be protective against the risk of dementia onset (23). This study consists of a relatively small number of participants−9,550 participants in total. The study also measured dementia with mixed definitions, including self-reported informant-reported scores above a certain threshold of IQCODE (Informant Questionnaire on Cognitive Decline in the Elderly). Instead, our study utilized the ICD-10 code definition of dementia with three categories–overall dementia, Alzheimer's disease, and vascular dementia. Consequently, unlike previous studies, our study presents compelling evidence that social trust in a community has a dementia-risk-reducing effect among older adults in the Korean population regardless of sex with an objective definition of dementia and a large study population.

Moreover, current literature suggests that depression is one of the influential determinants of dementia (Alzheimer's disease, vascular dementia, or any dementia) (24, 25). There has been substantial research regarding the association between social capital and depression as well. For instance, a cross-sectional study in China showed that social capital mediates depression (26). Another study from Indonesia proved that social trust is related to the severity of depressive symptoms (27). In this respect, it is vital to consider the possible confounding effect of depression on the association between social capital and the risk of dementia. In our study, this effect was considered by involving depression with ICD-10 diagnosis as one of the confounding variables. This makes our study more compelling that there is an association between social capital and the risk of dementia.

Recently, a cross-sectional study from Japan discovered that community-level social capital, including social trust, is associated with dementia (4). Although this study showed that higher community-level social trust is associated with a lower risk of dementia, it used the self-administered dementia checklist to measure subjective dementia symptoms. In other words, it used a limited definition to measure incident dementia. By contrast, our study provides more robust evidence than the previous study on the association between community-level social trust and the risk of dementia by having a direct measure of dementia. In addition, our study population was obtained from a nationwide cohort database, and the social trust value was calculated from a district representative sample and converted into the area-level value for all populations, which is different from the recent Japanese cross-sectional study that only consisted of 75,338 participants in total. In our study, a null association was discovered between social trust level and vascular dementia. However, though not statistically significant, there was an association with vascular dementia in our several results. Further studies considering enough vascular dementia cases are needed.

Various mechanisms have been proposed to explain the significant association of social capital, including community-level social trust, with dementia. First, social relationship engagement in districts with a high social trust may delay the incidence of dementia with cognitive stimulation and effective stress management by alleviating harmful stress nervous system responses (16). Second, the “use it or lose it” theory, which says that the brain can be considered as a muscle and various activities, including social activities that stimulate the brain (28), may be applied to explain the relationship between social trust and the risk of dementia. Reshadi (29) showed that social trust and social participation have a significant relationship. In this respect, participating more in social activities in districts with high social trust leads to frequent stimulation of the brain, which may delay the incidence of dementia. Third, low levels of social trust may increase social stress and anxiety, which may result in the stimulation of the hypothalamic-pituitary-adrenal axis (HPA axis) (15). The stimulation of the HPA axis leads to high blood cortisol levels, which can cause detrimental diseases such as dementia (30, 31). By decreasing social stress and anxiety, a high social trust may help reduce the risk of dementia. Finally, by promoting health awareness, communities with a higher community-level social trust may reduce unhealthy lifestyle behaviors (32). Instead of healthy lifestyle pathways, this study focuses on the psychosocial pathway, health service utilization pathway, and other possible undetermined mechanisms.

Several limitations were discovered in this study. First, the social trust index was calculated at one point so that the possible change in the social trust level at various points was not contemplated. This can be considered a minor limitation since communities in a country participate in the same social activities, which may affect the change in social trust, and it has been demonstrated that the trust values return to the primary levels (33). Second, our retrospective cohort study could be biased by omitted variables. The factors that we could not observe or measure in the database, not social trust level, may affect dementia risk. However, it is hard to control omitted variable bias in any retrospective database (34). Third, this study only considered participants living within seven metropolitan cities, without participants living within rural areas. Further studies may be needed to determine the influence of social trust on dementia in older adults within rural areas. Fourth, we could not consider all risk factors modeled by the 2020 *Lancet* Commission, like education level and air pollution (35), as covariates due to a lack of information. However, we accounted for income level, which might be a proxy for education level. Future studies that consider other risk factors, which were not included in our analysis, are required to validate our findings. Finally, since the NHIS database does not include imaging data and screening examinations, the operational definition of dementia was based on the ICD-10 codes and the prescription of dementia-related medications. Specifically, the severity and stage of dementia were not considered in the analyses due to the lack of information.

Despite these limitations, this study has significant strengths. Based on our information, this is the first study to investigate community-level social trust and dementia risk in Korea. Our study consists of a large cohort population of nearly 2 million participants with the minimal exclusion of the study population. Second, we excluded the area-level confounders such as urbanization by limiting the study population to seven metropolitan areas of the Republic of Korea. Third, our study's result was significant in both men and women. This is notable because some previous studies showed different results by gender of the study participants. Fourth, our study divides dementia into overall dementia, Alzheimer's disease, and vascular dementia with ICD-10 code diagnosis and prescription of anti-dementia drugs. Reliable medical claims records support the validation of dementia events. Lastly, we accounted for various confounding variables such as comorbidities in the analysis.

Findings in our study suggest that higher social trust at the community level is associated with a lower risk of dementia. The association is preserved with additional adjustments for dementia risk factors such as comorbidities, depression, diabetes, and cardiovascular disease. This proposes that social trust, as part of social capital, is a significant indicator of dementia. Therefore, having a health care policy that can improve community-level social trust may help reduce the risk of dementia, especially among individuals over 50 years of age.

## Data availability statement

The datasets are not publicly available as they are protected by the Korean National Health Insurance Service. Anonymized versions can be made available upon institutional approval. Further information is available via https://nhiss.nhis.or.kr and queries should be directed to the corresponding author(s).

## Ethics statement

The studies involving human participants were reviewed and approved by Seoul National University Hospital Institutional Review Board (IRB number: E-1806-076-951). Written informed consent for participation was not required for this study in accordance with the national legislation and the institutional requirements.

## Author contributions

Study concept and design: JH, SJP, J-KL, HJJ, and SMP. Acquisition of data: J-KL and SMP. Analysis and interpretation of data and critical revision of the manuscript for important intellectual content: JH, SJP, J-KL, HJJ, JO, SC, SJ, KHK, JSS, and SMP. Drafting of the manuscript: JH, SJP, J-KL, and SMP. Statistical analysis: JH and SJP. All authors contributed to the article and approved the submitted version.

## Funding

This research was supported by the Korea Disease Control and Prevention Agency (Grant Number: 2021-11-017). SJP received a scholarship from the BK21 FOUR education program from the National Research Foundation of Korea (NRF).

## Conflict of interest

The authors declare that the research was conducted in the absence of any commercial or financial relationships that could be construed as a potential conflict of interest.

## Publisher's note

All claims expressed in this article are solely those of the authors and do not necessarily represent those of their affiliated organizations, or those of the publisher, the editors and the reviewers. Any product that may be evaluated in this article, or claim that may be made by its manufacturer, is not guaranteed or endorsed by the publisher.
