# Supplementary material for: Association between community-level social trust and the risk of dementia: A retrospective cohort study in the Republic of Korea
Source: Front Public Health. 2022 Oct 6;10:913319. doi: 10.3389/fpubh.2022.913319 (PMC9582360; doi:10.3389/fpubh.2022.913319)
Supplement: Supplementary file 1 [file Data_Sheet_1.docx]

***Supplementary Material***

**Association between Community-Level Social Trust and the Risk of Dementia: A Retrospective Cohort Study in the Republic of Korea**

**Supplementary Table S1.** Hazard ratios for dementia per 1 interquartile range increase in the social trust index

**Supplementary Table S2.** Stratified analysis on the association of social trust with dementia according to depression, cardiovascular disease, and diabetes

**Supplementary Table S3.** Descriptive characteristics of participants who underwent health examinations

**Supplementary Table S4.** Descriptive characteristics of participants who did not undergo health examinations

**Supplementary Table S1.** Hazard ratios for dementia per 1 interquartile range increase in the social trust index

|  | **Overall dementia** | **Alzheimer’s disease** | **Vascular dementia** |
| --- | --- | --- | --- |
| Number of people | 1,974,944 | 1,974,944 | 1,974,944 |
| Events | 25,839 | 23,170 | 2,379 |
| aHR (95% CI) | 0.98 (0.96-0.99) | 0.98 (0.96-0.99) | 1.01 (0.96-1.06) |
| P-value | 0.005 | 0.003 | 0.670 |

The aHRs were calculated by Cox proportional hazards regression after adjustments for age, sex, area of residence, household income, Charlson comorbidity index, depression, cardiovascular disease, and diabetes.

Acronyms: aHR, adjusted hazard ratio; CI, confidence interval.

**Supplementary Table S2.** Stratified analysis on the association of social trust with dementia according to depression, cardiovascular disease, and diabetes

|  | aHR (95% CI) | | | | |  |
| --- | --- | --- | --- | --- | --- | --- |
|  | Social Trust (Quintiles) | | | | | P for trend |
|  | 1st (Lowest) | 2nd | 3rd | 4th | 5th (Highest) |  |
| **Overall dementia** |  |  |  |  |  |  |
| Previous depression |  |  |  |  |  |  |
| No | 1.00 (reference) | 0.90 (0.86-0.95) | 0.99 (0.94-1.03) | 0.95 (0.90-0.99) | 0.91 (0.86-0.95) | 0.020 |
| Yes | 1.00 (reference) | 0.94 (0.84-1.04) | 0.94 (0.85-1.04) | 0.96 (0.86-1.06) | 0.84 (0.75-0.94) | 0.048 |
| Previous cardiovascular disease |  |  |  |  |  |  |
| No | 1.00 (reference) | 0.90 (0.85-0.95) | 0.97 (0.92-1.03) | 0.94 (0.89-0.998) | 0.89 (0.83-0.94) | 0.010 |
| Yes | 1.00 (reference) | 0.93 (0.87-0.99) | 0.99 (0.92-1.05) | 0.96 (0.90-1.02) | 0.90 (0.84-0.97) | 0.150 |
| Previous diabetes |  |  |  |  |  |  |
| No | 1.00 (reference) | 0.90 (0.85-0.95) | 0.98 (0.93-1.04) | 0.92 (0.87-0.98) | 0.90 (0.84-0.95) | 0.080 |
| Yes | 1.00 (reference) | 0.92 (0.86-0.99) | 0.98 (0.92-1.04) | 0.99 (0.93-1.05) | 0.90 (0.84-0.97) | 0.044 |
| **Alzheimer’s disease** |  |  |  |  |  |  |
| Previous depression |  |  |  |  |  |  |
| No | 1.00 (reference) | 0.90 (0.85-0.94) | 0.96 (0.92-1.01) | 0.93 (0.88-0.98) | 0.90 (0.85-0.95) | 0.011 |
| Yes | 1.00 (reference) | 0.97 (0.87-1.08) | 0.94 (0.84-1.05) | 0.97 (0.87-1.09) | 0.86 (0.76-0.97) | 0.079 |
| Previous cardiovascular disease |  |  |  |  |  |  |
| No | 1.00 (reference) | 0.91 (0.85-0.97) | 0.96 (0.90-1.02) | 0.94 (0.89-1.003) | 0.89 (0.84-0.95) | 0.011 |
| Yes | 1.00 (reference) | 0.91 (0.85-0.98) | 0.96 (0.89-1.02) | 0.93 (0.87-0.995) | 0.90 (0.83-0.97) | 0.079 |
| Previous diabetes |  |  |  |  |  |  |
| No | 1.00 (reference) | 0.90 (0.85-0.96) | 0.97 (0.92-1.04) | 0.92 (0.87-0.98) | 0.91 (0.85-0.97) | 0.169 |
| Yes | 1.00 (reference) | 0.92 (0.86-0.99) | 0.95 (0.88-1.01) | 0.96 (0.90-1.03) | 0.89 (0.82-0.96) | 0.009 |
| **Vascular dementia** |  |  |  |  |  |  |
| Previous depression |  |  |  |  |  |  |
| No | 1.00 (reference) | 0.83 (0.71-0.97) | 1.06 (0.91-1.22) | 1.00 (0.86-1.17) | 0.94 (0.79-1.11) | 0.514 |
| Yes | 1.00 (reference) | 0.68 (0.47-0.97) | 0.92 (0.66-1.28) | 0.86 (0.61-1.22) | 0.70 (0.47-1.03) | 0.594 |
| Previous cardiovascular disease |  |  |  |  |  |  |
| No | 1.00 (reference) | 0.71 (0.59-0.87) | 0.92 (0.77-1.11) | 0.82 (0.67-0.99) | 0.84 (0.68-1.03) | 0.768 |
| Yes | 1.00 (reference) | 0.93 (0.75-1.15) | 1.18 (0.97-1.45) | 1.19 (0.97-1.46) | 0.97 (0.78-1.22) | 0.405 |
| Previous diabetes |  |  |  |  |  |  |
| No | 1.00 (reference) | 0.79 (0.66-0.96) | 0.997 (0.84-1.19) | 0.90 (0.74-1.08) | 0.86 (0.70-1.05) | 0.845 |
| Yes | 1.00 (reference) | 0.82 (0.65-1.02) | 1.08 (0.88-1.33) | 1.09 (0.89-1.35) | 0.95 (0.75-1.21) | 0.378 |

The aHRs were calculated by Cox proportional hazards regression after adjustments for age, sex, area of residence, household income, Charlson comorbidity index, depression, cardiovascular disease, and diabetes.

Acronyms: aHR, adjusted hazard ratio; CI, confidence interval.

**Supplementary Table S3.** Descriptive characteristics of participants who underwent health examinations

|  | Social Trust (Quintiles) | | | | | P value |
| --- | --- | --- | --- | --- | --- | --- |
|  | 1st (Lowest) | 2nd | 3rd | 4th | 5th (Highest) |  |
| Number of people | 215,765 | 218,755 | 226,827 | 227,860 | 230,390 |  |
| Age, years, mean (SD) | 55.38 (9.65) | 55.12 (9.49) | 55.37 (9.76) | 55.87 (9.85) | 54.99 (9.65) | <0.001 |
| Sex, N (%) |  |  |  |  |  | <0.001 |
| Men | 102,208 (47.37) | 104,877 (47.94) | 108,600 (47.88) | 108,782 (47.74) | 113,761 (49.38) |  |
| Women | 113,557 (52.63) | 113,878 (52.06) | 118,227 (52.12) | 119,078 (52.26) | 116,629 (50.62) |  |
| Area of residence, N (%) |  |  |  |  |  | <0.001 |
| Capital | 131,387 (60.89) | 83,128 (38.00) | 142,980 (63.03) | 76,244 (33.46) | 30,867 (13.40) |  |
| Metropolitan city | 84,378 (39.11) | 135,627 (62.00) | 83,847 (36.97) | 151,616 (66.54) | 199,523 (86.60) |  |
| Household income, quartiles, N (%) |  |  |  |  |  | <0.001 |
| 1st (highest) | 74,105 (34.35) | 82,250 (37.60) | 90,816 (40.04) | 84,077 (36.90) | 95,443 (41.43) |  |
| 2nd | 50,980 (23.63) | 50,515 (23.09) | 49,152 (21.67) | 53,670 (23.55) | 52,285 (22.69) |  |
| 3rd | 41,430 (19.20) | 39,023 (17.84) | 37,879 (16.70) | 40,109 (17.60) | 36,913 (16.02) |  |
| 4th (lowest) | 49,250 (22.83) | 46,967 (21.47) | 48,980 (21.59) | 50,004 (21.95) | 45,749 (19.86) |  |
| Charlson comorbidity index, N (%) |  |  |  |  |  | <0.001 |
| 0 | 30,360 (14.07) | 31,331 (14.32) | 33,239 (14.65) | 31,367 (13.77) | 32,477 (14.10) |  |
| 1 | 47,273 (21.91) | 48,868 (22.34) | 50,488 (22.26) | 48,456 (21.27) | 51,191 (22.22) |  |
| ≥ 2 | 138,132 (64.02) | 138,556 (63.34) | 143,100 (63.09) | 148,037 (64.97) | 146,722 (63.68) |  |
| Previous depression, N (%) |  |  |  |  |  | <0.001 |
| No | 195,619 (90.66) | 198,071 (90.54) | 204,867 (90.32) | 205,749 (90.30) | 208,228 (90.38) |  |
| Yes | 20,146 (9.34) | 20,684 (9.46) | 21,960 (9.68) | 22,111 (9.70) | 22,162 (9.62) |  |
| Previous cardiovascular disease, N (%) |  |  |  |  |  | <0.001 |
| No | 173,168 (80.26) | 176,421 (80.65) | 181,640 (80.08) | 181,075 (79.47) | 187,702 (81.47) |  |
| Yes | 42,597 (19.74) | 42,334 (19.35) | 45,187 (19.92) | 46,785 (20.53) | 42,688 (18.53) |  |
| Previous diabetes, N (%) |  |  |  |  |  | <0.001 |
| No | 166,829 (77.32) | 172,048 (78.65) | 177,680 (78.33) | 175,575 (77.05) | 181,987 (78.99) |  |
| Yes | 48,936 (22.68) | 46,707 (21.35) | 49,147 (21.67) | 52,285 (22.95) | 48,403 (21.01) |  |

P value calculated by Chi-squared test for categorical variables and analysis of variance for continuous variables.

Acronyms: SD, standard deviation; N, number of people.

**Supplementary Table S4.** Descriptive characteristics of participants who did not undergo health examinations

|  | Social Trust (Quintiles) | | | | | P value |
| --- | --- | --- | --- | --- | --- | --- |
|  | 1st (Lowest) | 2nd | 3rd | 4th | 5th (Highest) |  |
| Number of people | 178,349 | 164,932 | 180,443 | 170,780 | 160,843 |  |
| Age, years, mean (SD) | 55.71 (11.42) | 55.37 (11.18) | 55.71 (11.42) | 56.18 (11.56) | 55.60 (11.48) | <0.001 |
| Sex, N (%) |  |  |  |  |  | <0.001 |
| Men | 87,999 (49.34) | 79,141 (47.98) | 87,233 (48.34) | 82,597 (48.36) | 75,493 (46.94) |  |
| Women | 90,350 (50.66) | 85,791 (52.02) | 93,210 (51.66) | 88,183 (51.64) | 85,350 (53.06) |  |
| Area of residence, N (%) |  |  |  |  |  | <0.001 |
| Capital | 111,770 (62.67) | 73,836 (44.77) | 124,388 (68.93) | 66,716 (39.07) | 24,799 (15.42) |  |
| Metropolitan city | 66,579 (37.33) | 91,096 (55.23) | 56,055 (31.07) | 104,064 (60.93) | 136,044 (84.58) |  |
| Household income, quartiles, N (%) |  |  |  |  |  | <0.001 |
| 1st (highest) | 53,010 (29.72) | 54,211 (32.87) | 63,852 (35.39) | 52,969 (31.02) | 55,672 (34.61) |  |
| 2nd | 40,350 (22.62) | 36,324 (22.02) | 37,666 (20.87) | 37,207 (21.79) | 35,135 (21.84) |  |
| 3rd | 37,860 (21.23) | 31,325 (18.99) | 32,280 (17.89) | 31,988 (18.73) | 27,753 (17.25) |  |
| 4th (lowest) | 47,129 (26.43) | 43,072 (26.12) | 46,645 (25.85) | 48,616 (28.47) | 42,283 (26.29) |  |
| Charlson comorbidity index, N (%) |  |  |  |  |  | <0.001 |
| 0 | 45,875 (25.72) | 40,732 (24.70) | 45,558 (25.25) | 41,790 (24.47) | 37,880 (23.55) |  |
| 1 | 39,550 (22.18) | 37,436 (22.70) | 40,071 (22.21) | 37,086 (21.72) | 36,074 (22.43) |  |
| ≥ 2 | 92,924 (52.10) | 86,764 (52.61) | 94,814 (52.55) | 91,904 (53.81) | 86,889 (54.02) |  |
| Previous depression, N (%) |  |  |  |  |  | <0.001 |
| No | 163,142 (91.47) | 150,100 (91.01) | 163,929 (90.85) | 155,174 (90.86) | 145,927 (90.73) |  |
| Yes | 15,207 (8.53) | 14,832 (8.99) | 16,514 (9.15) | 15,606 (9.14) | 14,916 (9.27) |  |
| Previous cardiovascular disease, N (%) |  |  |  |  |  | <0.001 |
| No | 145,744 (81.72) | 134,775 (81.72) | 146,138 (80.99) | 137,541 (80.54) | 132,116 (82.14) |  |
| Yes | 32,605 (18.28) | 30,157 (18.28) | 34,305 (19.01) | 33,239 (19.46) | 28,727 (17.86) |  |
| Previous diabetes, N (%) |  |  |  |  |  | <0.001 |
| No | 140,512 (78.78) | 131,597 (79.79) | 143,090 (79.30) | 133,155 (77.97) | 127,912 (79.53) |  |
| Yes | 37,837 (21.22) | 33,335 (20.21) | 37,353 (20.70) | 37,625 (22.03) | 32,931 (20.47) |  |

P value calculated by Chi-squared test for categorical variables and analysis of variance for continuous variables.

Acronyms: SD, standard deviation; N, number of people.
